# Supplementary material for: Infection-derived lipids elicit an immune deficiency circuit in arthropods
Source: Nat Commun. 2017 Feb 14;8:14401. doi: 10.1038/ncomms14401 (PMC5316886; doi:10.1038/ncomms14401)
Supplement: Supplementary Information — Supplementary Figures and Supplementary Tables [file ncomms14401-s1.pdf]

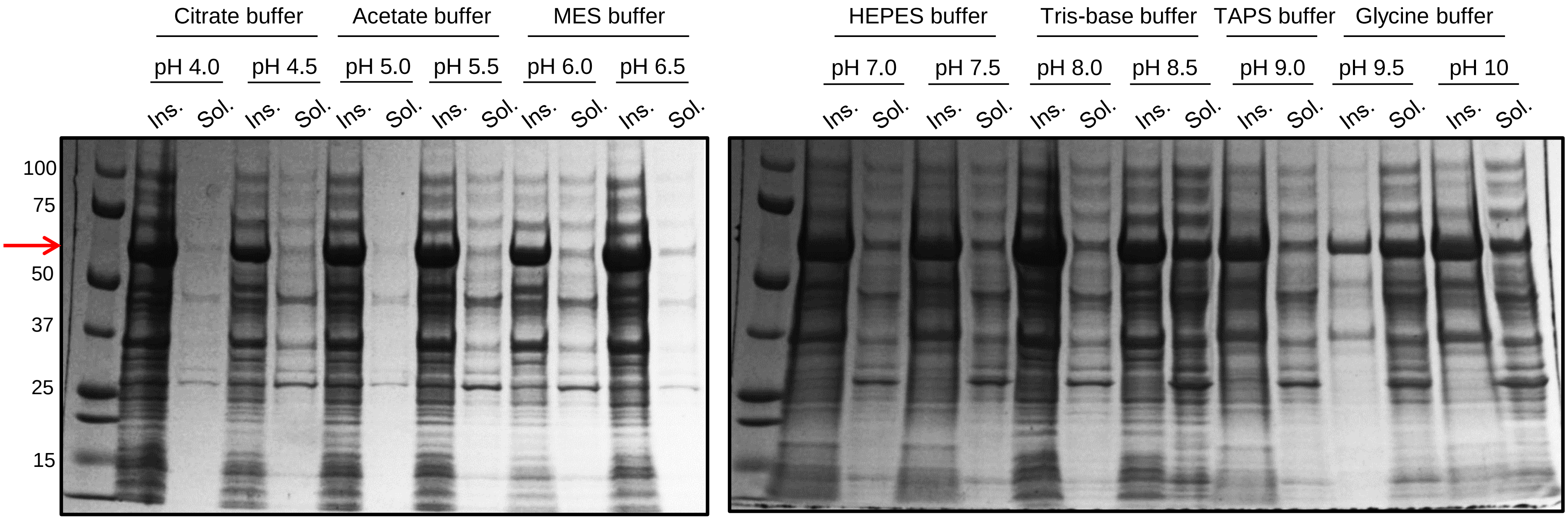

Supplementary Figure 1 (Related to Figure 1). XIAP protein expression in *E. coli*. *E. coli* was grown overnight in LB medium and induced at 20°C in the presence of 0.1 mM IPTG. *E. coli* was lysed (buffers pH 4-10, as indicated) and separated by centrifugation to determine protein solubility. Ins – Insoluble; Sol – Soluble.

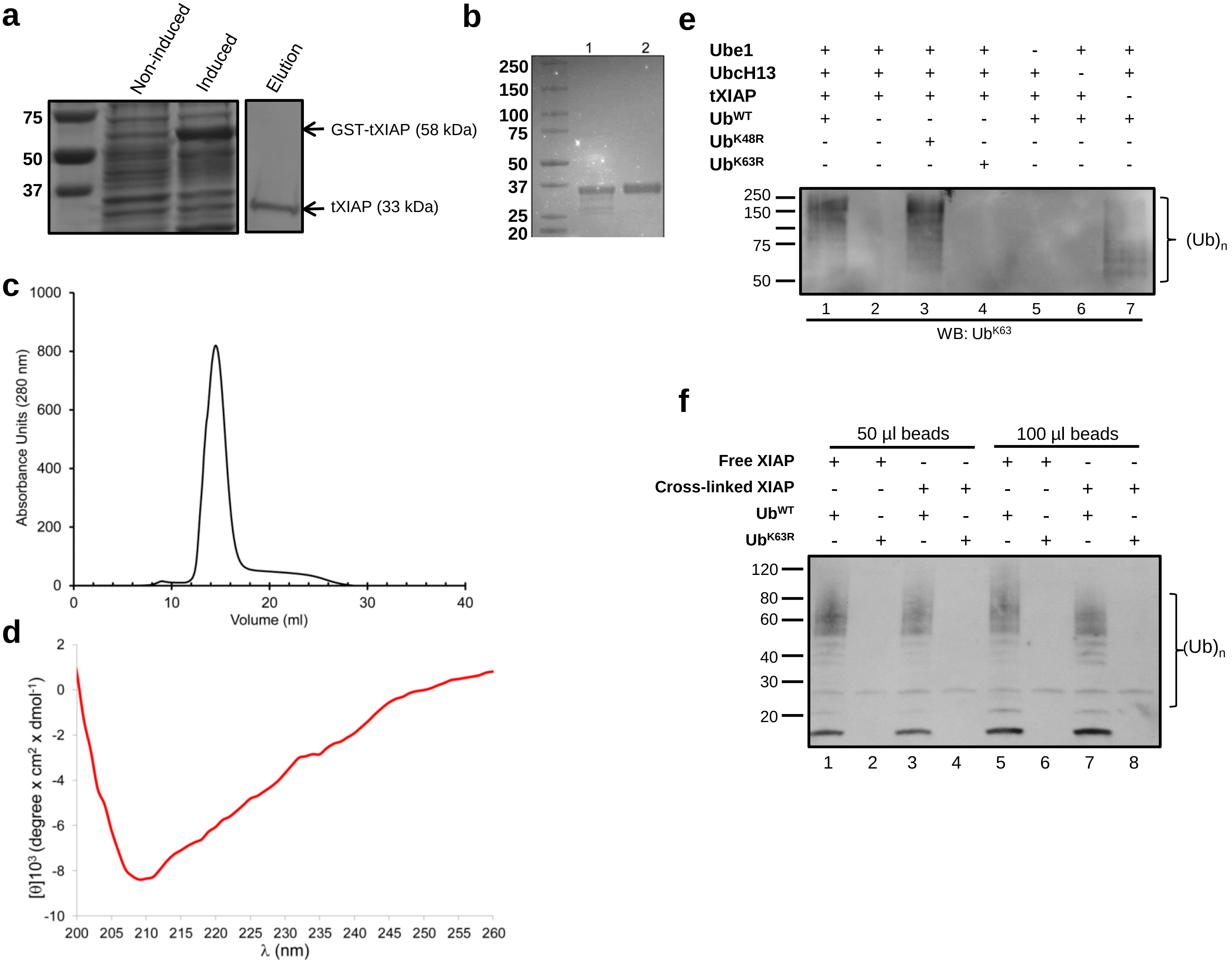
a
Non-induced
Induced
Elution
75
50
37
GST-tXIAP (58 kDa)
tXIAP (33 kDa)
b
1
2
250
150
100
75
50
37
25
20
c
Absorbance Units (280 nm)
Volume (ml)
d
[θ]10³ (degree x cm² x dmol⁻¹)
λ (nm)
e
Ube1
UbcH13
tXIAP
UbWT
UbK48R
UbK63R
(Ub)n
WB: UbK63
f
50 µl beads
100 µl beads
Free XIAP
Cross-linked XIAP
UbWT
UbK63R
(Ub)n

Supplementary Figure 2 (Related to Figure 1). *I. scapularis* produces K63-linked polyubiquitin chains (**a**) The *I. scapularis* XIAP protein fused to the GST-tag (GST-tXIAP) was induced following *E. coli* growth overnight. The GST tag was removed and XIAP was eluted. (**b**) XIAP was expressed and lysed in Tris-EDTA buffer (pH 8.9), purified using an agarose column following GST tag removal with the "PreScission" protease (1) and size exclusion in the FPLC instrument (2). (**c**) Elution peaked with the retention time of 18 minutes. (**d**) Verification of the XIAP secondary structure by circular dichroism. (**e**) XIAP polyubiquitylation assays in the presence (Lane 1) or absence (Lane 2) of wild-type ubiquitin (Ub^{WT}), ubiquitin carrying a lysine to arginine mutation in the position 48 (Ub^{K48R}) (Lane 3), ubiquitin carrying a lysine to arginine mutation in the position 63 (Ub^{K63R}) (Lane 4). Assays were also performed in the absence of E1 (Ube1) (Lane 5), E2 (UbcH13) (Lane 6) or XIAP (Lane 7), respectively. Aliquots were resolved in 12% SDS-PAGE and then probed against Ub^{K63}-specific antibodies (WB: UB^{K63}). (**f**) Assays showing K63-polyubiquitylation in free (Lanes 1-2, 5-6) and covalently-linked XIAP-GST (Lanes 3-4, 7-8) bound to glutathione-agarose columns.

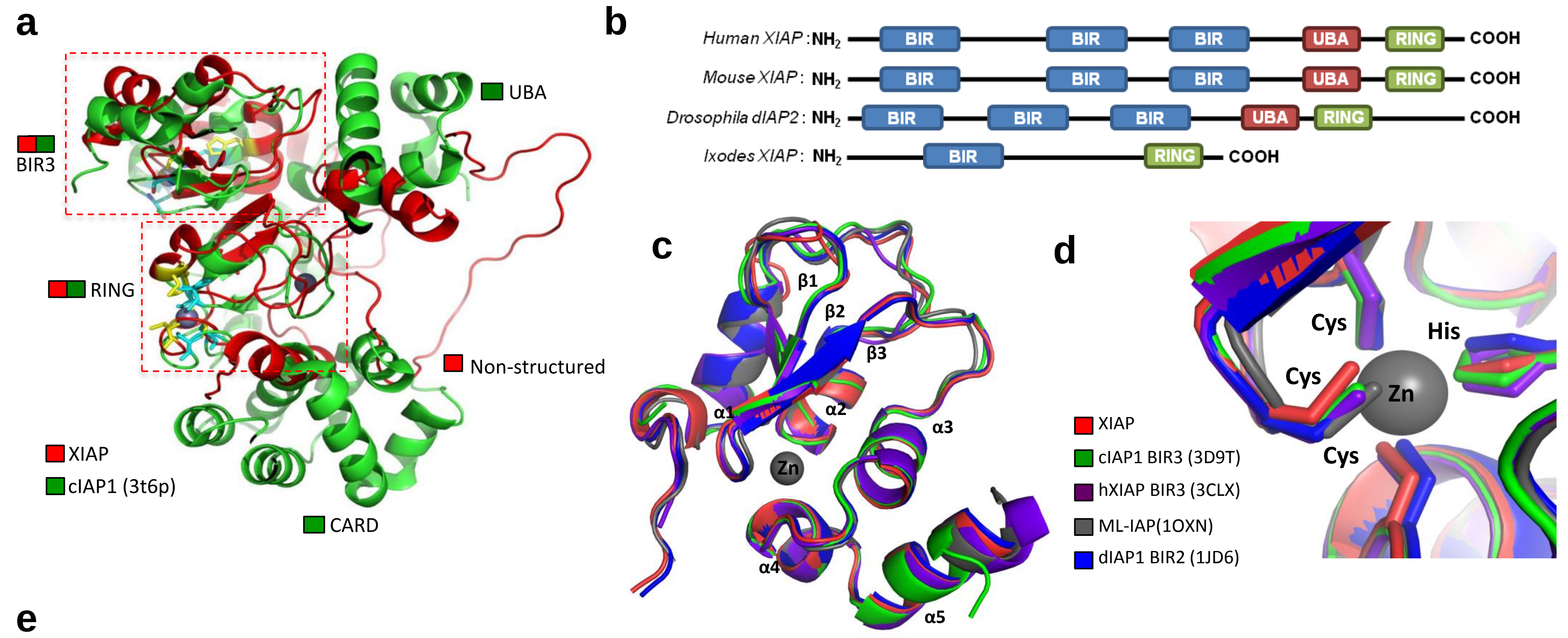

e

Type	Name	Alias	Position	α-helix 4								
I	dIAP2 (BIR 1)		12-77	V	A	E	R	H	R	R	S	S
I	BIRC4 (BIR 1)	XIAP	29-94	A	V	G	R	H	R	K	V	S
I	BIRC2 (BIR 1)	cIAP2	32-97	P	T	E	K	H	K	K	L	Y
I	BIRC3 (BIR 1)	cIAP1	49-114	P	I	Q	K	H	K	Q	L	Y
II	dIAP2 (BIR 2)		116-180	A	F	E	E	H	K	R	F	F
II	BIRC4 (BIR 2)	XIAP	166-231	A	W	S	E	H	R	R	H	F
II	BIRC2 (BIR 2)	cIAP2	172-236	A	M	S	E	H	L	R	H	F
II	BIRC3 (BIR 2)	cIAP1	187-251	A	M	S	E	H	R	R	H	F
II	BIRC5	Survivin	18-88	P	I	E	E	H	K	K	H	S
II	BIRC6	BRUCE	289-359	P	W	S	E	H	E	R	H	S
II	dIAP1 (BIR 2)	Thread	47-111	P	V	P	E	H	Q	R	W	S
III	BIRC1 (BIR 2)	NAIP	162-228	P	W	K	E	H	A	K	W	F
III	dIAP2 (BIR 3)		215-280	P	W	F	E	H	A	K	W	S
III	dIAP1 (BIR 1)	Thread	229-294	P	W	E	Q	H	A	L	W	L
III	BIRC2 (BIR 3)	cIAP2	258-323	P	W	V	Q	H	A	K	W	F
III	BIRC4 (BIR 3)	XIAP	268-331	P	W	E	Q	H	A	K	W	Y
III	BIRC3 (BIR 3)	cIAP1	272-337	P	W	V	E	H	A	K	W	F
**III**	**XIAP**		**66-134**	**P**	**F**	**E**	**E**	**H**	**A**	**R**	**W**	**Y**
III	BIRC8	ILP2	7-70	P	W	E	Q	H	A	K	W	Y
III	BIRC7	ML-IAP	90-155	P	W	T	E	H	A	K	W	F
X	BIRC1 (BIR 3)	NAIP	281-346	P	L	D	D	H	T	R	C	F
X	BIRC1 (BIR 1)	NAIP	68-128	P	I	E	D	H	K	R	F	H

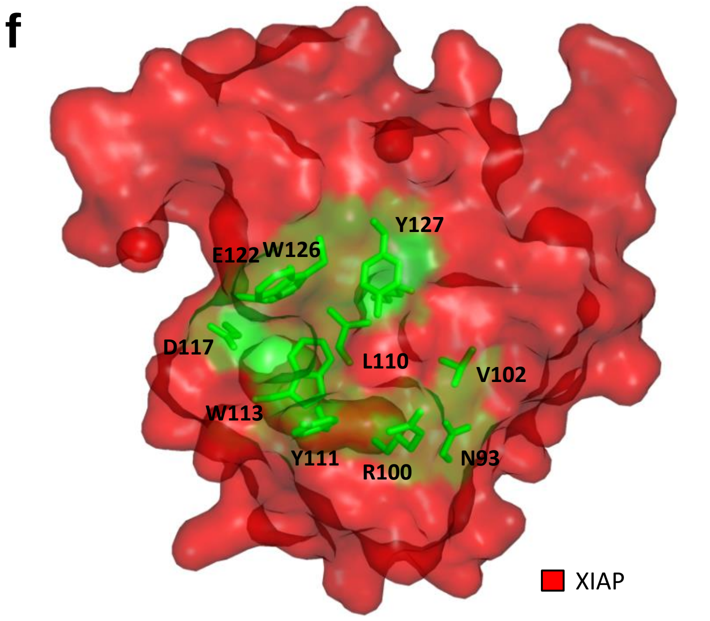

Supplementary Figure 3 (Related to Figure 1). *I. scapularis* XIAP tertiary structure. (**a**) Thread of XIAP onto the cIAP1 structure (PDB:3T6P), depicting the conserved BIR and RING domains (dashed red lines). The BIR and RING structures of XIAP highlights one and two zinc molecules with side chains in stick representation. Cyan and yellow side chains represent conserved amino acids that are involved in Zn atom coordination. The superimposition of cIAP1 (green) shows the absence of a known CARD or UBA domain in the *I. scapularis* XIAP protein (red). A non-structured region in XIAP is also shown. (**b**) *I. scapularis* XIAP domain comparison with human XIAP, mouse XIAP and *Drosophila* dIAP2. Baculoviral IAP repeat domain (BIR; blue), ubiquitin associated domain (UBA; red) and Really Interesting New Gene domain (RING; green) (**c**) Cartoon depiction of the least squares structural alignment of the tick XIAP (tXIAP) BIR domain and proteins identified from the Dali server: (1) cIAP1 BIR3 (PDB:3D9T); (2) human XIAP (hXIAP) BIR3 (PDB:3CLX); (3) ML-IAP (PDB:1OXN); (4) dIAP1 BIR2 (PDB:1JD6). (**d**) Conserved Zinc (Zn) coordinating residues in the XIAP BIR domain are shown. (**e**) Alignment of human and *Drosophila* IAP BIR domains showing the residues that depict the IBM groove (grey residues) when compared to tick XIAP (highlighted in bold red). The type and position of BIR domains at indicated proteins are also shown. (**f**) Surface rendition of the modeled *I. scapularis* XIAP BIR domain. The tick XIAP BIR model depicting the peptide binding pocket is displayed in green with the side chains in stick representation. See also Supplementary Figures 1-2.

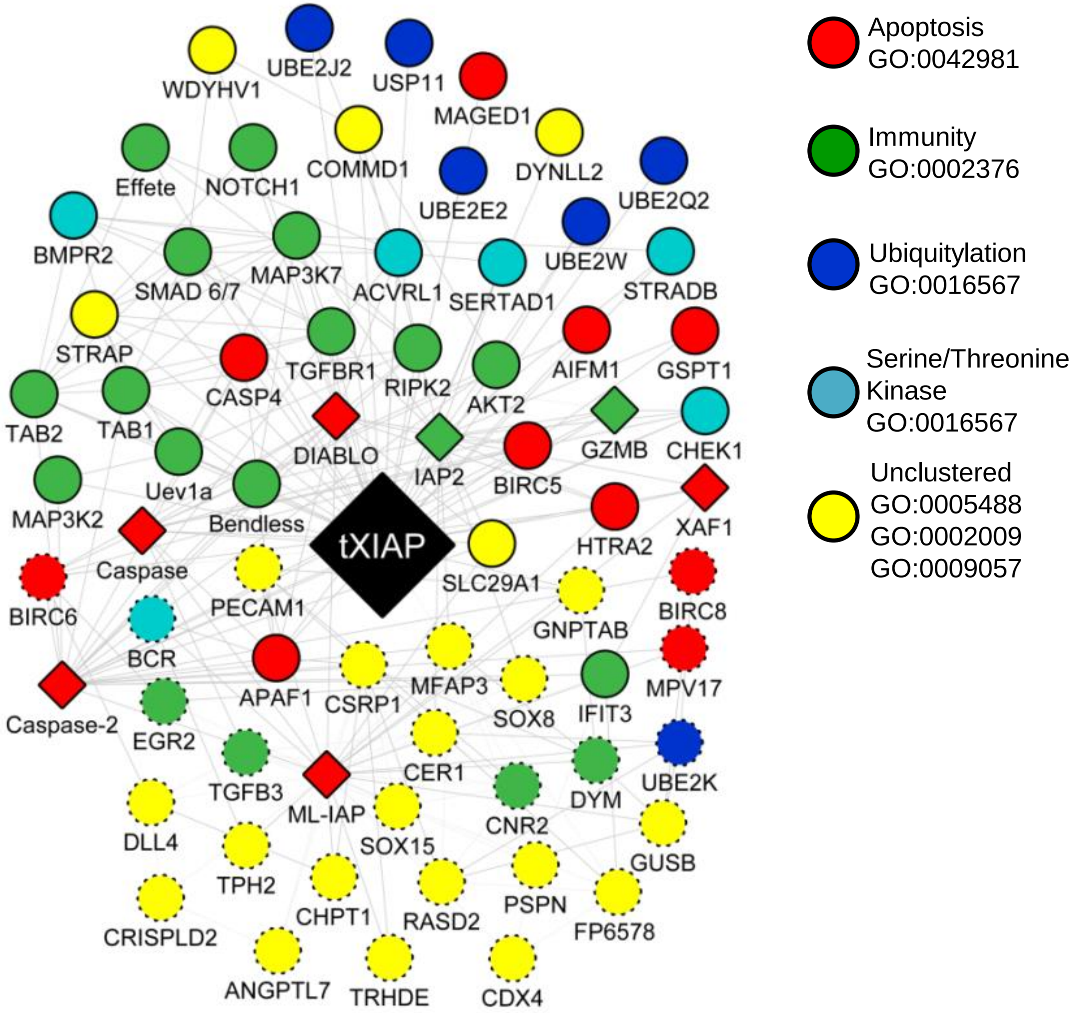

Supplementary Figure 4 (Related to Figure 1 and Supplementary Table 1). Extended *I. scapularis* XIAP interactome. Human XIAP or ML-IAP interactome was compiled according to GeneMANIA and Cytoscape. *I. scapularis* homologues were obtained based on PSI-BLAST and GeneCard searches. Sixty eight candidates were grouped according to the GO functional categories available at DAVID. Circles carrying solid and dotted borders indicate candidates originated from either the human XIAP or ML-IAP query, respectively. Diamond nodes indicate human homologue proteins common to both interactomes.

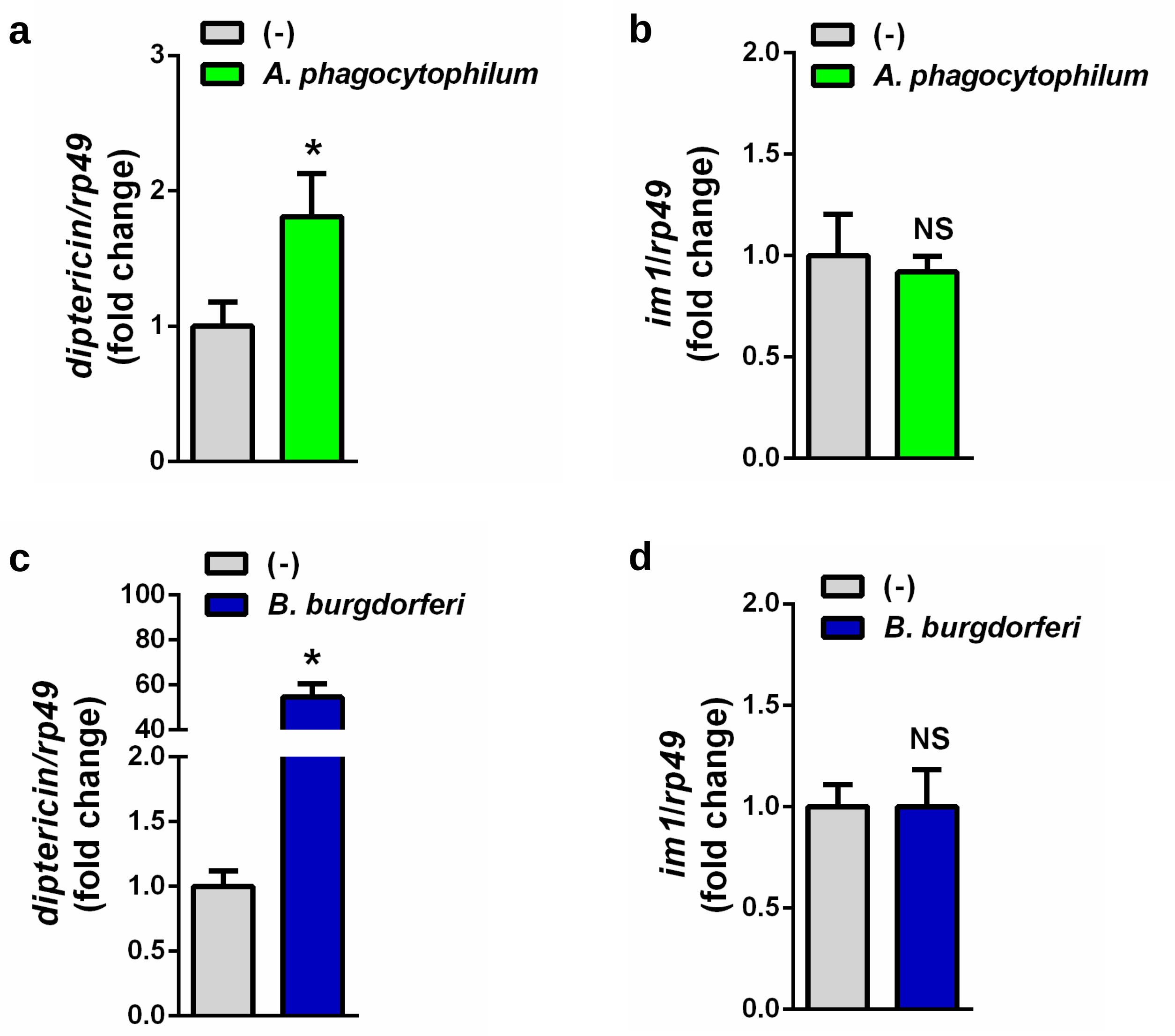

Supplementary Figure 5 (Related to Figure 5 and Supplementary Table 2). *B. burgdorferi* and *A. phagocytophilum* elicit the IMD pathway in *Drosophila.* Triplicate samples of 1x10^6 S2* cells were primed with ecdysone (1 µM) and infected with (**a**-**b**) *A. phagocytophilum* or (**c**-**d**) *B. burgdorferi* at MOI 50. Pathway stimulation was measured by quantifying *diptericin* (IMD) and *im1* (Toll) transcripts. Three biological replicates and two technical replicates are represented as the mean ± standard errors of the means (SEM). Student's t test. *$P < 0.05$. NS – not significant. (-), non-stimulated.

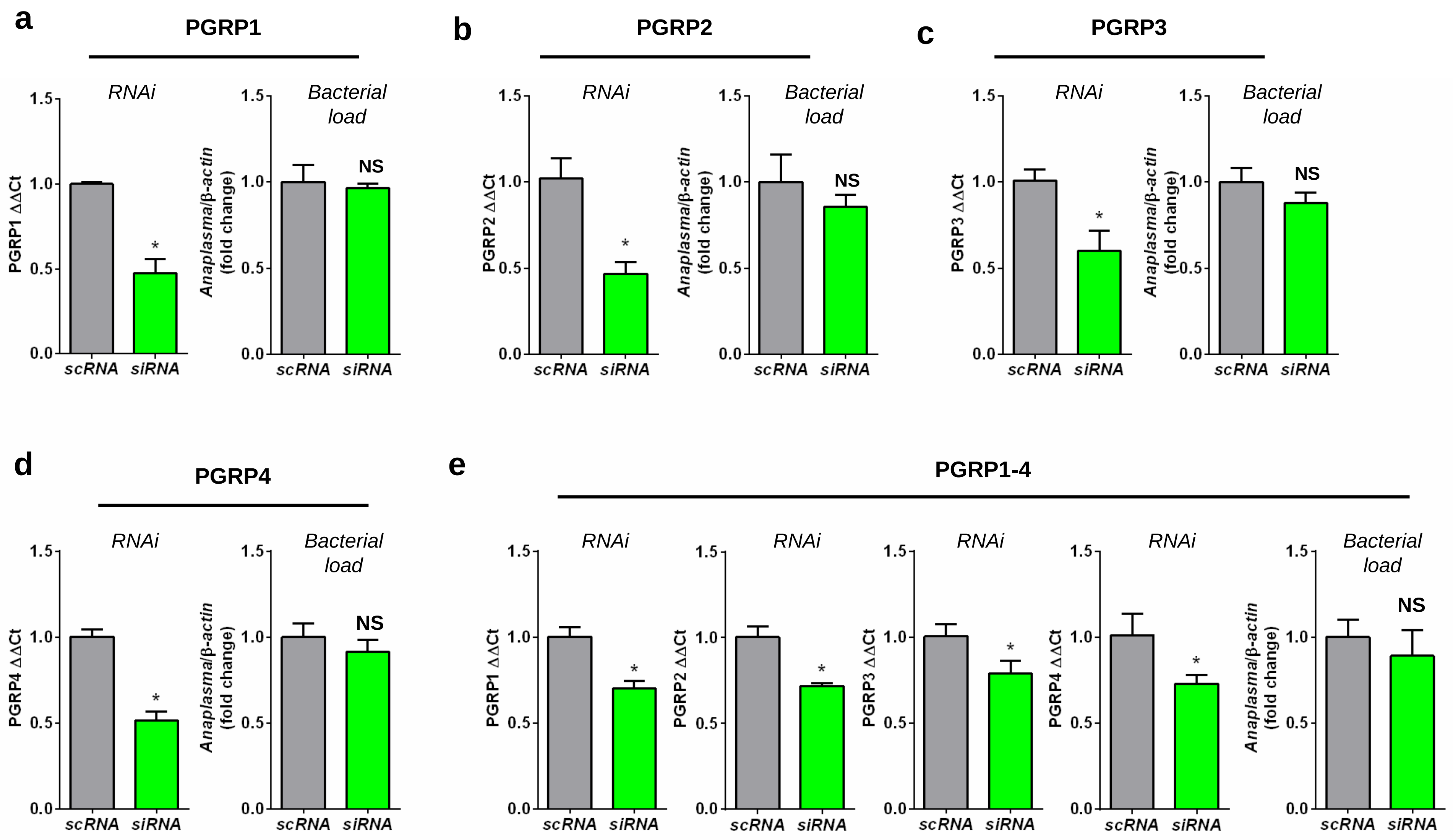

Supplementary Figure 6 (Related to Figure 6 and Supplementary Tables 2 and 3). Soluble *I. scapularis* PGRPs do not impact the IMD pathway during *A. phagocytophilum* infection. (**a**-**d**) Five replicates of ISE6 (1x 10^5) cells were infected with *A. phagocytophilum* (MOI 50) following targeted gene silencing of (**a**) PGRP-1 (**b**) PGRP-2 (**c**) PGRP-3 (**d**) PGRP-4 and (**e**) PGRP-1-4. Gene silencing and *A. phagocytophilum* load (16s rDNA*)* were measured by qRT-PCR at 18 hours post-infection in ISE6 cells. Three biological replicates and two technical replicates are represented as the mean ± standard errors of the means (SEM). Student's t test. **P* < 0.05. scRNA – scrambled RNA; siRNA – small interference RNA. See also Supplementary Table 2.

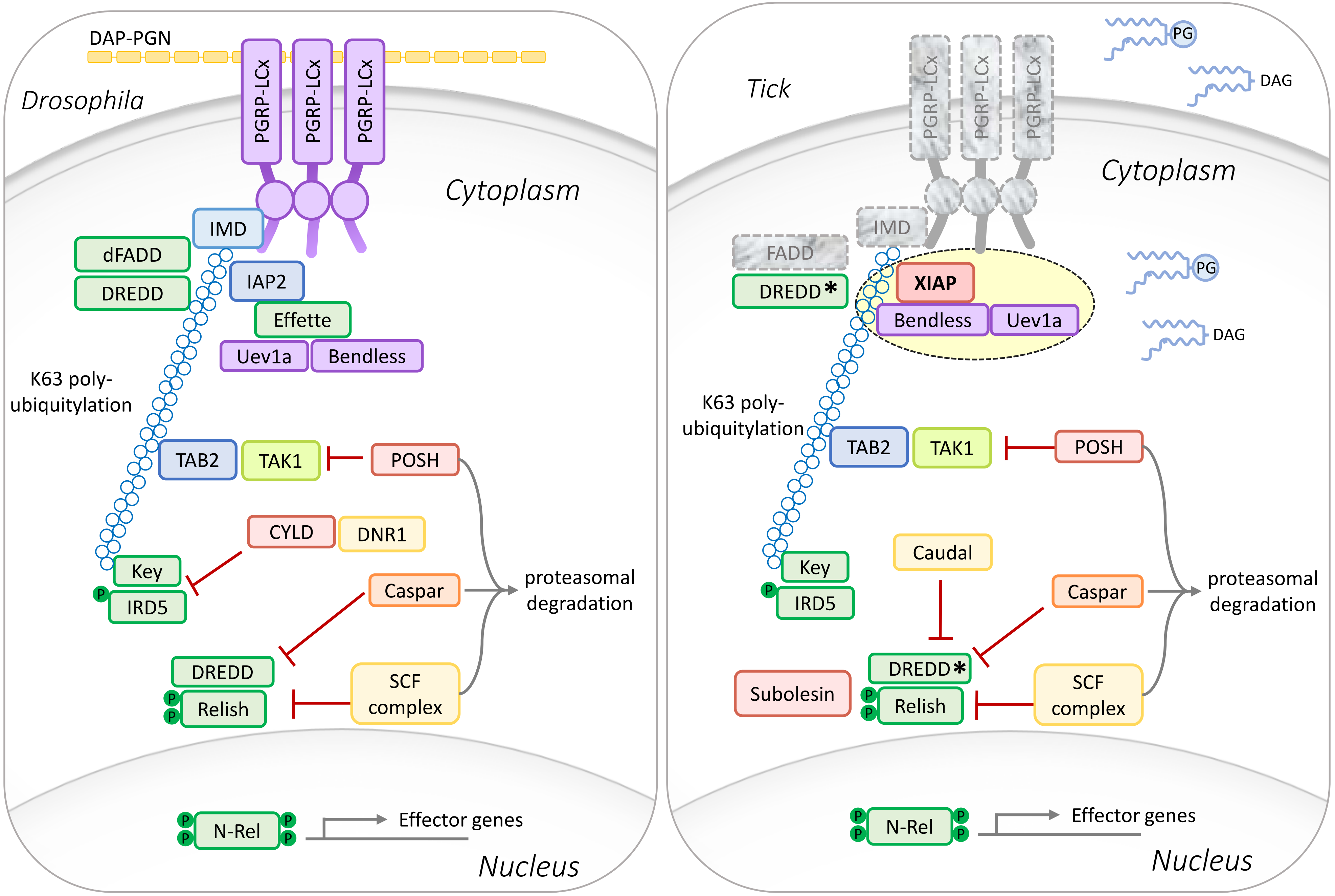
DAP-PGN
Drosophila
PGRP-LCx
PGRP-LCx
PGRP-LCx
Cytoplasm
IMD
dFADD
DREDD
IAP2
Effette
Uev1a
Bendless
K63 poly-
ubiquitylation
TAB2
TAK1
POSH
CYLD
DNR1
Key
P
IRD5
Caspar
proteasomal
degradation
DREDD
P
P
Relish
SCF
complex
P
P
N-Rel
P
P
Effector genes
Nucleus
Tick
PGRP-LCx
PGRP-LCx
PGRP-LCx
PG
DAG
Cytoplasm
IMD
FADD
DREDD*
XIAP
Bendless
Uev1a
PG
DAG
K63 poly-
ubiquitylation
TAB2
TAK1
POSH
Key
P
IRD5
Caudal
Caspar
proteasomal
degradation
Subolesin
DREDD*
P
P
Relish
SCF
complex
P
P
N-Rel
P
P
Effector genes
Nucleus

Supplementary Figure 7 (Related to Figure 7). Schematic representation of the tick IMD pathway when compared to insects (**a**) The insect IMD pathway senses and binds DAP-type peptidoglycan from Gram-negative bacteria via peptidoglycan recognition proteins LC (PGRP-LC) and PGRP-LE. Adapter molecules IMD and Fas-Associated protein with Death Domain (FADD) are recruited to the RHIM domains of PGRP-LC and engage the caspase-8 homologue DREDD. DREDD cleaves IMD, exposing a lysine (K)63-polyubiquitylation binding site. The E3 ubiquitin ligase Inhibitor of Apoptosis Protein (IAP)2 together with three E2 ubiquitin conjugating enzymes, Uev1a, Bendless and Effete, poly-ubiquitylate IMD. The ubiquitin scaffold leads to the recruitment of TGF-β (TAK1) and Tak1-binding protein 2 (TAB2) and the I-κB kinase (IKK) complex, which phosphorylates the NF-κB transcription factor, Relish. Upon phosphorylation, the N-terminal domain of Relish (N-Rel) is cleaved by DREDD and is translocated to the nucleus, driving the production of anti-microbial peptides. (**b**) Multiple homologues associated with this pathway are found in ticks. However, notable exceptions include transmembrane PGRP-LC, IMD and FADD. *the presence of DREDD has not been demonstrated in ticks; however, other caspases are available in the *I. scapularis* genome. The location of either POPG or PODAG during stimulation is unknown. XIAP-Bendless interaction is highlighted in yellow and dotted lines. Faded molecules in the IMD pathway have not been identified in the *I. scapularis* genome.

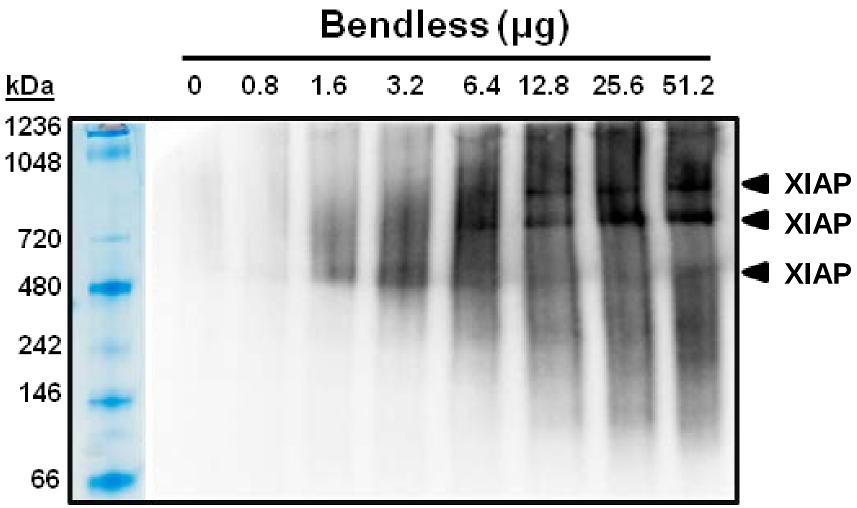

Supplementary Figure 8 (Related to Figure 2c). Uncropped Western blot of XIAP gel-shift assay. Native gel analysis of a fixed amount of recombinant (0.2 μg) XIAP incubated with increasing amounts of recombinant Bendless. Immunoblotted (WB) with anti-XIAP. The WB shown is one of two biological replicates.

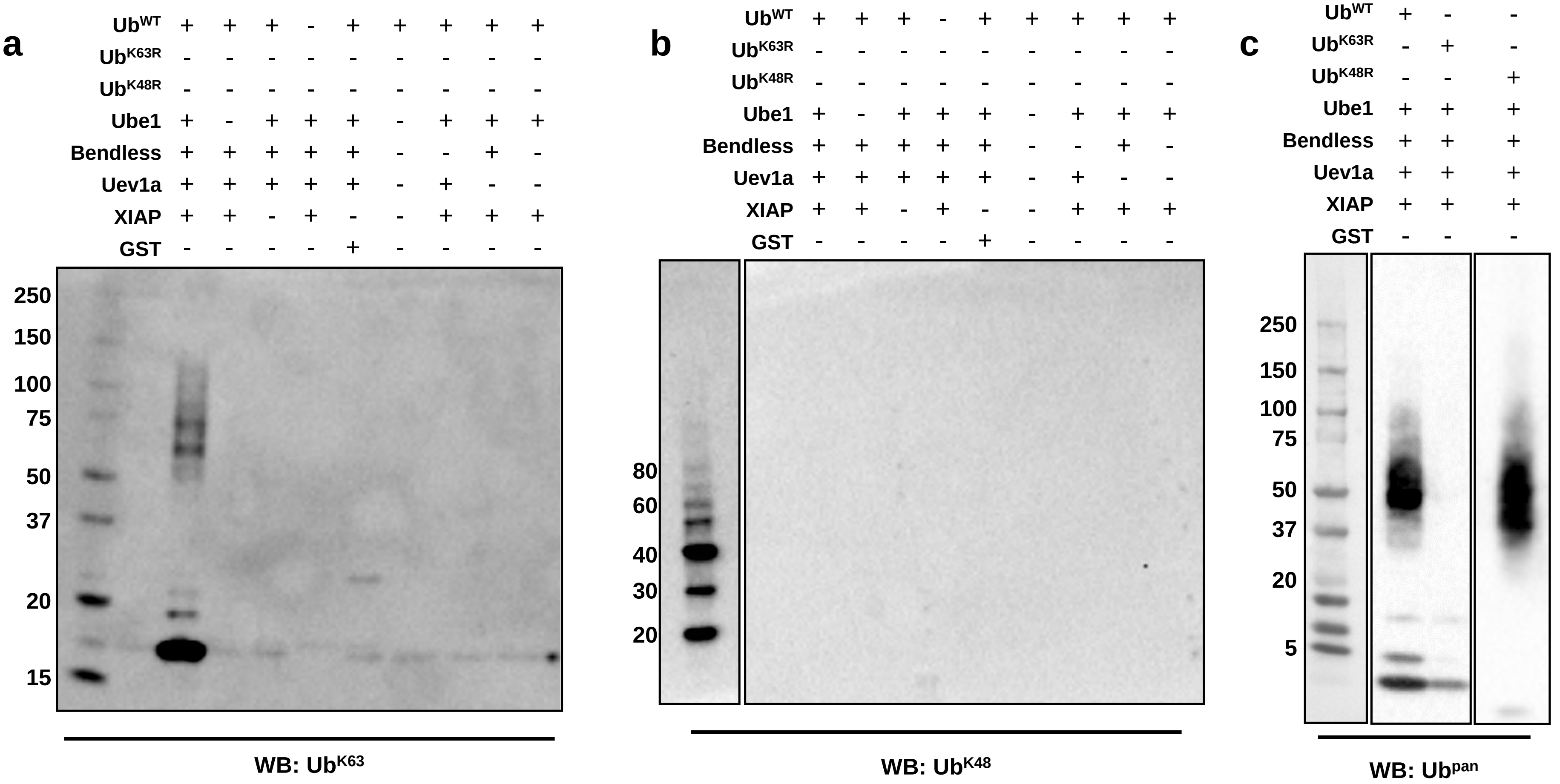

Supplementary Figure 9 (Related to Figure 2f). Uncropped Western blot of XIAP polyubiquitylation assays. XIAP polyubiquitylation assay with recombinant Bendless (Lane 1). Control conditions were performed in the absence of an E1 enzyme – Ube1 (Lane 2), XIAP (Lane 3, 5 and 6), wild type ubiquitin (Lane 4, 11 and 12) and Bendless (Lanes 6, 7 and 9) and Uev1a (Lanes 6, 8-9). Immunoblots (WB) were probed with antibodies specific for (**a**) K63 and (**b**) K48 polyubiquitin chains or with (**c**) a pan-ubiquitin antibody. GST was used as a negative control. WB shown represent one of two biological replicates.

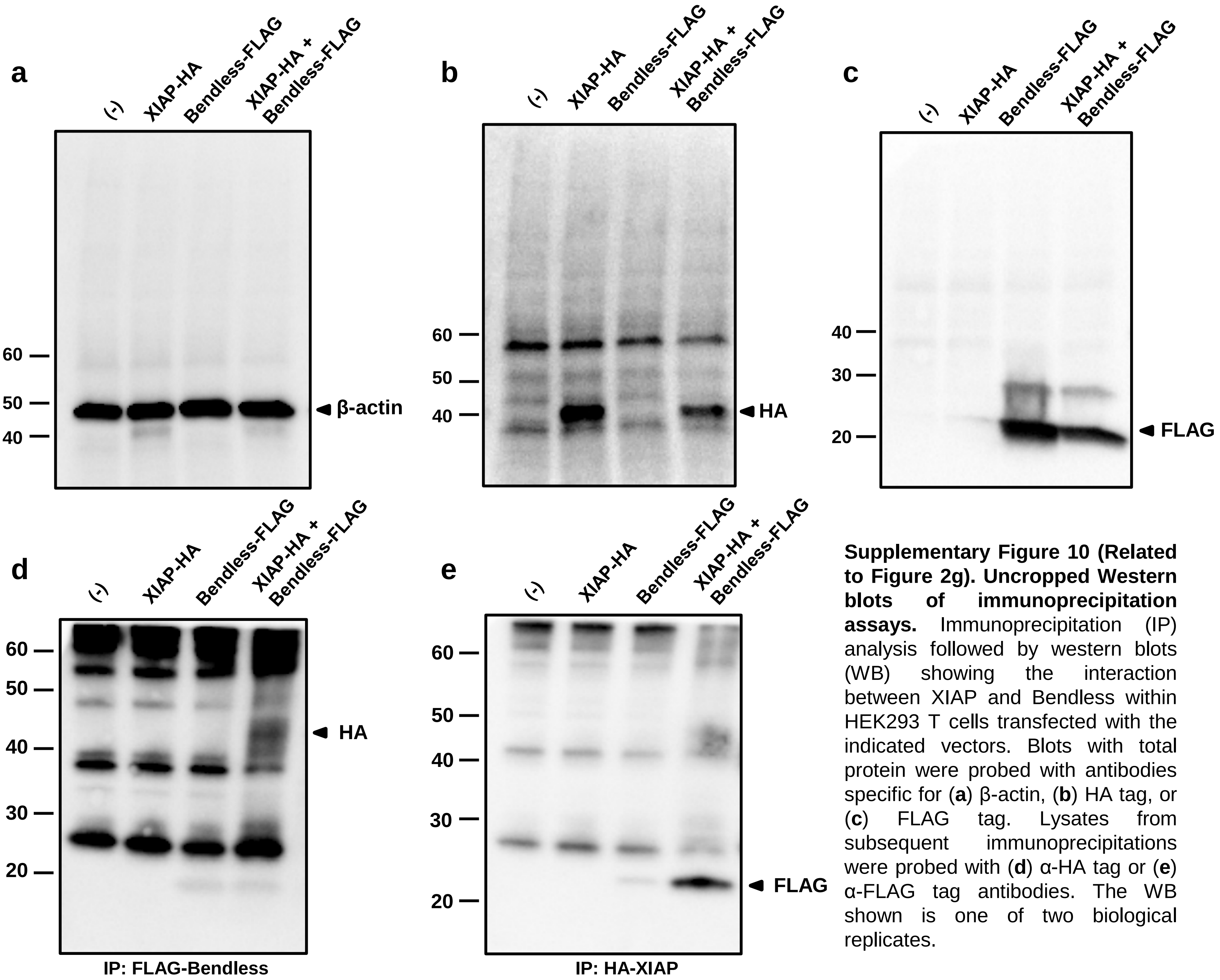

Supplementary Figure 10 (Related to Figure 2g). Uncropped Western blots of immunoprecipitation assays. Immunoprecipitation (IP) analysis followed by western blots (WB) showing the interaction between XIAP and Bendless within HEK293 T cells transfected with the indicated vectors. Blots with total protein were probed with antibodies specific for (**a**) β-actin, (**b**) HA tag, or (**c**) FLAG tag. Lysates from subsequent immunoprecipitations were probed with (**d**) α-HA tag or (**e**) α-FLAG tag antibodies. The WB shown is one of two biological replicates.

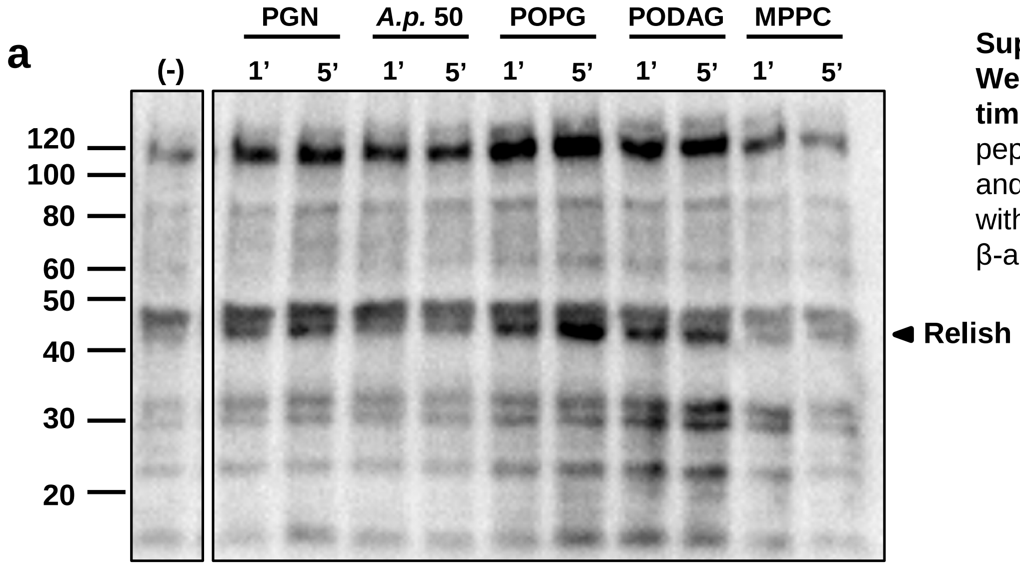

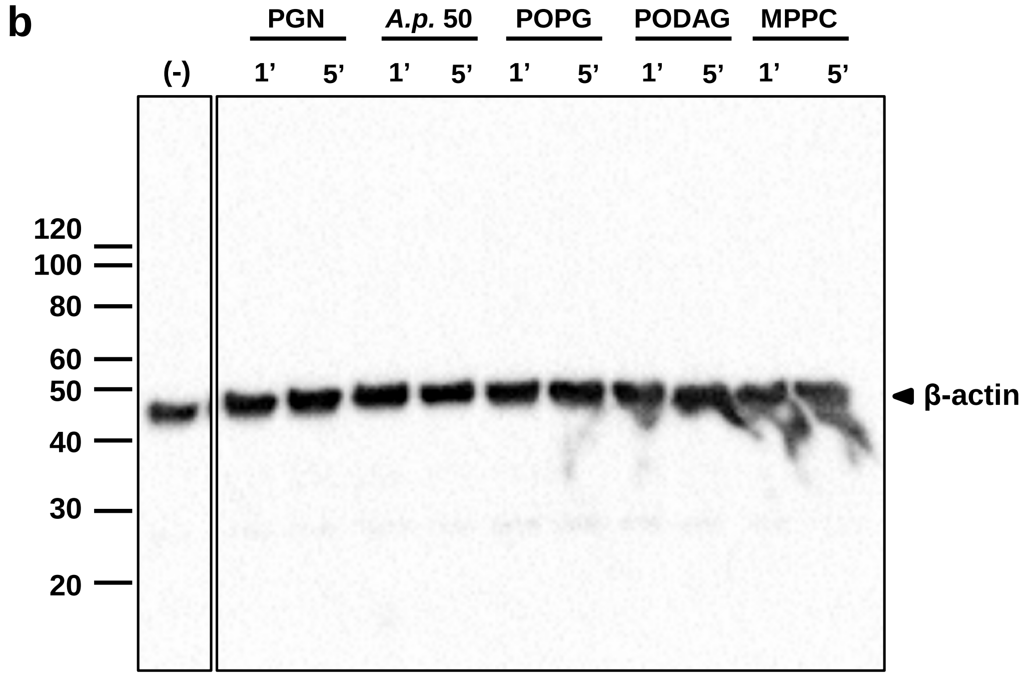

Supplementary Figure 11 (Related to Figure 5f). Uncropped Western blots (WB) of ISE6 cells stimulated at indicated time points. Unstimulated (-), diaminopimelic-type peptidoglycan (PGN) (10 µg/ml), *A. phagocytophilum* (MOI 50) and indicated lipids (1 ng) at 1 and 5 minutes. Blots were probed with antibodies specific for (**a**) rabbit polyclonal α-Relish or (**b**) α-β-actin. The WB shown is one of three biological replicates.

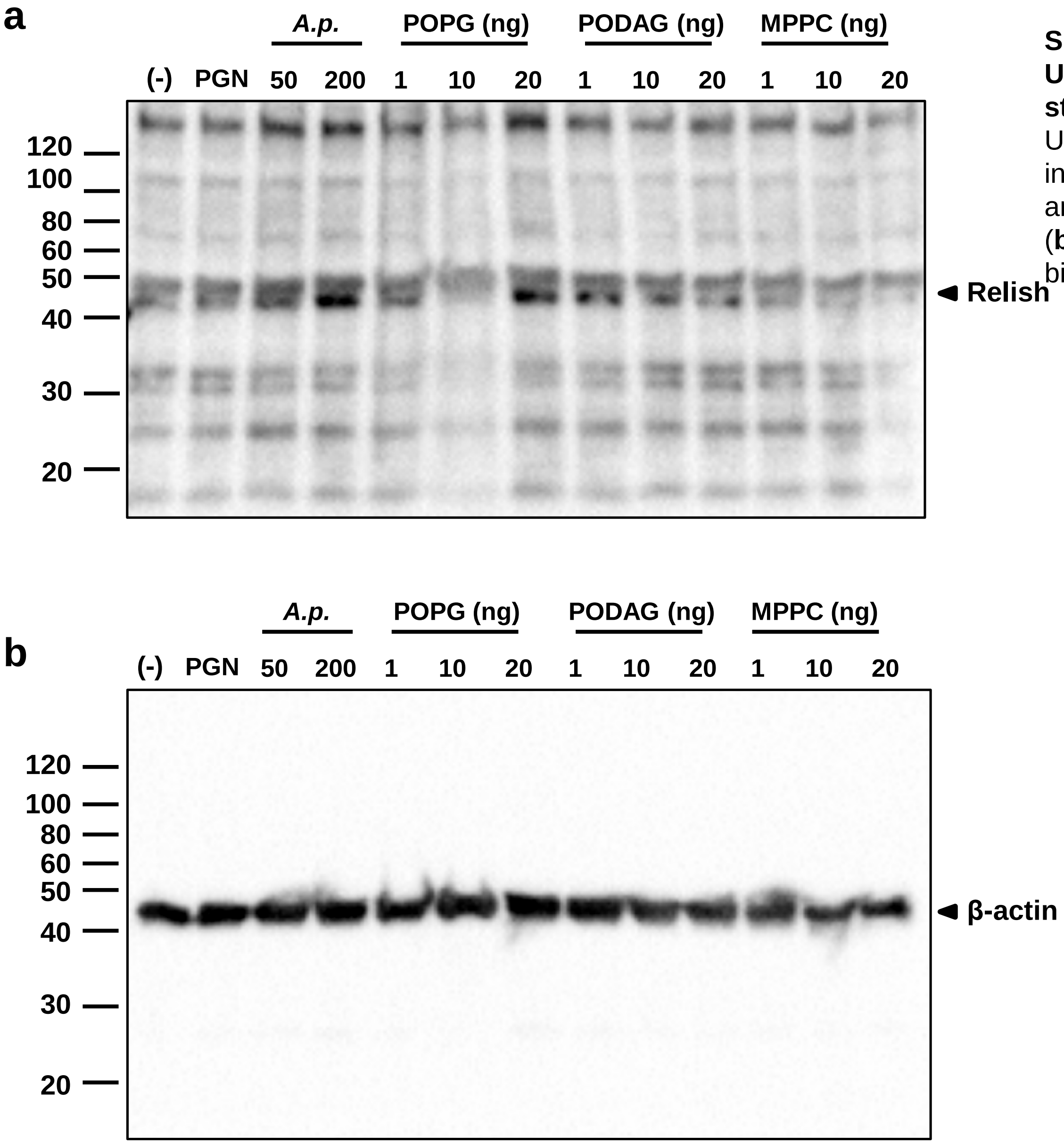

Supplementary Figure 12 (Related to Figure 5g). Uncropped Western blots (WBs) of ISE6 cells stimulated at indicated concentrations. Unstimulated (-), *A. phagocytophilum* (MOI 50) and indicated lipids (1 ng). Blots were probed with antibodies specific for (**a**) rabbit polyclonal α-Relish or (**b**) α-β-actin. The WB shown is one of three biological replicates.

Supplementary Table 1 (Related to Figure 1 and Supplementary Figure 4). The tick XIAP interactome derived from the human XIAP and ML-IAP integrated network

Tick Name	Tick Accession	Ref Seq Protein	Human Accession	Human Name	E Value	% Query Coverage	% Identity
UBE2Q2	B7P1N9	XP_002433447.1	Q8WVN8	UBE2Q2	7.00E-142	99	52
CNR2	B7P3B3	XP_002403852.1	P34972	CNR2	1.00E-07	86	24
TPH2	B7P4Z5	XP_002406648.1	Q8IWU9	TPH2	0.0	86	65
FP6578	B7P5P3	XP_002407783.1	Q6ZRI6	FP6578	1.00E-04	29	39
DIABLO	B7P5Y4	XP_002408096.1	Q9NR28	DIABLO	9.00E-16	63	28
GNPTAB	B7P614	XP_002408193.1	Q3T906	GNPTAB	2.00E-125	64	44
SLC29A1	B7P9P6	XP_002405059.1	Q99808	SLC29A1	2.00E-57	98	34
APAF1	B7PB18	XP_002407629.1	O14727	APAF1	2.00E-115	99	27
CSRP1	B7PC19	XP_002409223.1	P21291	CSRP1	4.00E-49	98	48
WDYHV1	B7PD34	XP_002410596.1	Q96HA8	WDYHV1	8.00E-59	99	44
COMMD1	B7PDX0	XP_002399524.1	Q8N668	COMMD1	2.00E-41	90	37
IFIT3	B7PDX5	XP_002399529.1	O14879	IFIT3	4.00E-04	58	26
XIAP	B7PF95	XP_002433867.1	P98170	XIAP	1.00E-26	84	26
XIAP	B7PF95	XP_002433867.1	Q96CA5	BIRC7	5.00E-30	82	29
XIAP	B7PF95	XP_002433867.1	Q96P09	BIRC8	1.00E-24	97	28
AKT2	B7PHG1	XP_002402787.1	P31751	AKT2	6.00E-153	80	58
GUSB	B7PJE8	XP_002407819.1	P08236	GUSB	1.00E-70	40	47
DYNLL2	B7PJZ9	XP_002408929.1	Q96FJ2	DYNLL2	2.00E-65	100	96
Bendless	B7PKK7	XP_002400703.1	P61088	UBE2N	1.00E-101	98	89
Effete	B7PKN3	XP_002434331.1	P51668	UBED1	1.00E-101	100	87
Effete	B7PKN3	XP_002434331.1	P61077	UBED3	4.00E-105	100	93
GSPT1	B7PLA6	XP_002434554.1	P15170	GSPT1	0.0	91	75
BIRC6	B7PLJ5	XP_002434643.1	Q9NR09	BIRC6	9.00E-34	5	43
AIFM1	B7PLJ7	XP_002434645.1	O95831	AIFM1	2.00E-135	66	54
SOX15	B7PLL5	XP_002434663.1	O60248	SOX15	6.00E-07	34	32
PSPN	B7PME5	XP_002434943.1	O60542	PSPN	1.00E-09	57	27
CHPT1	B7PMX3	XP_002435121.1	Q8WUD6	CHPT1	1.00E-131	92	49
UBE2K	B7PN10	XP_002435158.1	P61086	UBE2K	3.00E-101	99	76
MEKK3	B7PND3	XP_002435273.1	Q9Y2U5	MEKK3	3.00E-56	46	38
Uev1a	B7PP20	XP_002435512.1	Q13404	UBEV1	4.00E-76	98	86
Uev1a	B7PP20	XP_002435512.1	Q15819	UBEV2	1.8	11	24
SERTAD1	B7P9B6	XP_002403854.1	Q9UHV2	SERTAD1	1.00E-04	29	42
RASD2	B7PPF1	XP_002435643.1	Q96D21	RASD2	2.00E-42	92	41
TRHDE	B7PQN4	XP_002436076.1	Q9UKU6	TRHDE	7.00E-145	86	31
CRISPLD2	B7PQW1	XP_002436153.1	Q9H0B8	CRISPLD2	2.00E-21	41	30
STRAP	B7PS00	XP_002401479.1	Q9Y3F4	STRAP	5.00E-21	82	25
NOTCH1	B7PT11	XP_002403554.1	P46531	NOTCH1	0.0	98	46
UBE2W	B7PTB2	XP_002404101.1	Q96B02	UBE2W	2.00E-84	90	77
TGFB3	B7PV78	XP_002407531.1	P10600	TGFB3	1.00E-21	92	25

TAB1	B7PWS4	XP_002410221.1	Q15750	TAB1	2.00E-39	67	35
EGR2	B7PXK1	XP_002401004.1	P11161	EGR2	3.00E-68	40	79
SOX8	B7PYW8	XP_002404127.1	P57073	SOX8	3.00E-36	16	77
XAF1	B7PZ96	XP_002404975.1	Q6GPH4	XAF1	8.00E-04	52	31
ANGPTL7	B7PZB3	XP_002405085.1	O43827	ANGPTL7	4.00E-44	65	39
PECAM1	B7PZR6	XP_002405927.1	P16284	PECAM1	2.00E-11	74	20
HTRA2	B7PZV8	XP_002406208.1	O43464	HTRA2	2.00E-46	45	43
TAB2	B7Q0C5	XP_002407435.1	Q9NYJ8	TAB2	2.00E-26	39	37
UBE2E2	B7Q173	XP_002408959.1	Q96LR5	UBE2E2	3.00E-108	86	83
CHEK1	B7Q2N1	XP_002410911.1	O14757	CHEK1	2.00E-49	58	35
RIPK2	D9IQE8	ADK23790.1	O43353	RIPK2	2.00E-71	56	22
BMPR2	B7Q593	XP_002401624.1	Q13873	BMPR2	2.00E-148	42	50
CER1	B7Q6G3	XP_002402983.1	O95813	CER1	5.00E-10	32	31
IAP2	B7Q7F6	XP_002403970.1	Q13075	BIRC1	6.00E-40	30	30
IAP2	B7Q7F6	XP_002403970.1	Q13489	BIRC2	5.00E-63	70	30
IAP2	B7Q7F6	XP_002403970.1	Q13490	BIRC3	2.00E-67	73	29
SMAD6/7	B7Q8J0	XP_002405164.1	O15105	SMAD7	1.00E-22	80	33
SMAD6/7	B7Q8J0	XP_002405164.1	O43541	SMAD6	4.00E-26	64	31
UBE2J2	B7Q8X9	XP_002405482.1	Q8N2K1	UBE2J2	2.00E-82	65	62
CDX4	B7Q9V0	XP_002406414.1	O14627	CDX4	1.00E-20	27	47
Caspase-2	B7QAN7	XP_002412613.1	P42575	CASP2	9.00E-21	68	26
Caspase-2	B7QAN7	XP_002412613.1	P55211	CASP8	2.00E-27	62	30
Caspase-2	B7QAN7	XP_002412613.1	Q14790	CASP9	5.00E-30	60	29
TGFBR1	B7QAV0	XP_002412676.1	P36897	TGFBR1	0.0	93	63
ACVRL1	B7P561	XP_002407058.1	P37023	ACVRL1	2.00E-81	92	26
BCR	B7QB94	XP_002412820.1	P11274	BCR	8.00E-143	67	35
GZMB	B7QD33	XP_002413447.1	P10144	GZMB	3.00E-34	92	31
USP11	B7QDB1	XP_002413525.1	P51784	USP11	0.0	90	41
BIRC5	B7QE45	XP_002413809.1	O15392	BIRC5	2.00E-42	69	55
Caspase	B7QF31	XP_002414145.1	P42574	CASP3	9.00E-59	83	40
Caspase	B7QF31	XP_002414145.1	P55210	CASP7	3.00E-63	83	42
Caspase	B7QF31	XP_002414145.1	P55212	CASP6	5.00E-53	87	38
MAGED1	B7QGP1	XP_002400239.1	Q9Y5V3	MAGED1	5.00E-23	17	45
MPV17	B7QGS9	XP_002400740.1	P39210	MPV17	8.00E-38	94	40
CASP4	B7QHN6	XP_002414693.1	P49662	CASP4	6.00E-06	31	29
DLL4	B7QHQ8	XP_002414716.1	Q9NR61	DLL4	3.00E-144	80	40
STRADB	B7QJ15	XP_002415172.1	Q9C0K7	STRADB	3.00E-06	73	19
MAP3K7	B7QK24	XP_002415531.1	O43318	MAP3K7	4.00E-75	60	84
MFAP3	B7QKH9	XP_002415684.1	P55082	MFAP3	3.00E-08	19	38
DYM	B7QN16	XP_002400517.1	Q7RTS9	DYM	2.00E-137	77	45

Supplementary Table 2 Primers used

Name	Target gene	Primer sequence
Anaplasma phagocytophilum 16S (qRT-PCR)	NC_007797	F 5’-CAGCCACACTGGAACTGAGA-3'<br>R 5'-CCCTAAGGCCTTCCTCACTC-3'
Ixodes scapularis Actin (qRT-PCR)	AF426178	F 5’-GGTATCGTGCTCGACTC-3’<br>R 5’-ATCAGGTAGTCGGTCAGG-3’
Ixodes scapularis XIAP_qPCR	XM_002433822	F 5’- CAGAGCAATGGACAGCCTTT-3’<br>R 5’- CTCTGGATCCCCCTTGAACT-3’
Ixodes scapularis Bendless_qPCR	XM_002400659	F 5’-CCGTTACTTTCCGCAGACTT-3’<br>R 5’-TACAAGACCGTCACGCACTC-3’
Ixodes scapularis Uev1a_qPCR	XM_002435467.1	F 5’-ATGACCTTGACCCACTGGAC-3’<br>R 5’-CTCATTGATGCCTCCCATCT-3’
Ixodes scapularis Caspar_qPCR	XM_002405982.1	F 5’-CGACATCCGAGCGGCCATTC-3’<br>R 5’-CCTCGTCCGACACGTTAGC-3’
Ixodes scapularis Relish_qPCR	GBBN01020499.1[a]	F 5’-AGAATGTCCGCCACCGTTTTTTTCTGC-3’<br>R 5’-CACGTGCACCGCCTCACCATGAAGG-3’
Ixodes scapularis PGRP1_qPCR	XM_002411731.1	F 5’-CCAGTATCGTTCGCCACTG-3’<br>R 5’-GCTCCAATGGCACCAAAG-3’
Ixodes scapularis PGRP2_qPCR	XM_002433644.1	F 5’-GGCAAGACTACCACATGG-3’<br>R 5’- CCGATGCCATCAAAGCC -3’
Ixodes scapularis PGRP3_qPCR	XM_002410377.1	F 5’- CCGCCATGCAGCTCATC -3’<br>R 5’- GGAAGGCTTCTCCTGGG -3’
Ixodes scapularis PGRP4_qPCR	XM_002413046.1	F 5’- CGGTGTTGACATGTGCCG -3’<br>R 5’- CACGTAGGGAACTCGGGC -3’
Ixodes scapularis MyD88_qPCR	XM_002407328.1	F 5’-CCTGAGCCACTGGAGC-3’<br>R 5’-CGCAAGCCGAAGAGCAC-3’
Ixodes scapularis JAK_qPCR	XM_002406395.1	F 5’-GAGTACCTGGAAGAGAAG-3’<br>R 5’-TGTGGATGTAGTAGTAGTG-3’
Drosophila Diptericin_qPCR	NM_057460.4	F 5’-CCGCAGTACCCACTCAATCT-3’<br>R 5’-ACTGCAAAGCCAAAACCATC-3’
Drosophila IM1_qPCR	NM_137475.3	F 5’- TCCACTGTCGCCCGATCC -3’<br>R 5’- CTTGGGTTGAAACTTCCTACTTGC -3’
Drosophila Rp49_qPCR	NM_001144655.3	F 5’- AAGCTAGCCCAACCTGGTTG -3’<br>R 5’- GTGCGCTTCTTCACGATCT -3’
Ixodes scapularis Isc2_relish	GBBN01020499.1[a]	F 5’- AGAATGTCCGCCACCGTTTTTTTCTGC -3’<br>R 5’- GCTACTCTGGCTGAACGGGCTGAACCG -3’
Ixodes scapularis XIAP_siRNA_619	XM_002433822	F 5'-AAGCTACTCATCTCTCGAGGTCCTGTCTC-3'<br>R 5'-AAACCTCGAGAGATGAGTAGCCCTGTCTC-3'
Ixodes scapularis XIAP_scrambled	N/A	F 5’-AAGTCGCTACAGTCCGTTACTCCTGTCTC-3’<br>R 5’-AAAGTAACGGACTGTAGCGACCCTGTCTC-3’
Ixodes scapularis Bendless_siRNA_69	XM_002400659	F 5’-AAGCCTAATACTACTAAATCACCTGTCTC-3’<br>R 5’-AATGATTTAGTAGTATTAGGCCCTGTCTC-3’
Ixodes scapularis Bendless_scrambled	N/A	F 5’-AAAGCATCGTGTTTGGAGCTTTCCTGTCTC-3’<br>R 5’-AATACGCATAATACTACATCCCTGTCTC-3’
Ixodes scapularis Uev1a_siRNA_251	XM_002435467.1	F 5’-AAGCATCAGATTCATTTCCAACCTGTCTC-3’<br>R 5’-AATTGGAAATGAATCTGATGCCCTGTCTC-3’
Ixodes scapularis Uev1a_scrambled	N/A	F 5’-AAATTCGCTTAACTACGTAACCCTGTCTC-3’<br>R 5’-AAGTTACGTAGTTAAGCGAATCCTGTCTC-3’
Ixodes scapularis Caspar_siRNA_430	XM_002405982.1	F 5’-AAGATCTTCCTCCCCTATCTACCTGTCTC-3’<br>R 5’-AATAGATAGGGGAGGAAGATCCCTGTCTC-3’
Ixodes scapularis Caspar_scrambled	N/A	F 5’-AAGCCTCGCAGACTTAACCAACCTGTCTC-3’<br>R 5’-AATTGGTTAAGTCTGCGAGGCCCTGTCTC-3’
Ixodes scapularis Relish_siRNA_54	GBBN01020499.1[a]	F 5’-AACCTCACACCATTTGCCTTTCCTGTCTC-3’<br>R 5’-AAAAAGGCAAATGGTGTGAGGCCTGTCTC-3’
Ixodes scapularis Relish_scrambled	N/A	F 5’-AAGACGCCTATCGCCTACATTCCTGTCTC-3’<br>R 5’-AAAATGTAGGCGATAGGCGTCCCTGTCTC-3’
Ixodes scapularis PGRP1_siRNA_42	XM_002411731.1	F 5’-AACCAGTTATGTGGAACCTATCCTGTCTC-3’<br>R 5’-AAATAGGTTCCACATAACTGGCCTGTCTC-3’
Ixodes scapularis	N/A	F 5’-AAGTGGAATATCCGCATCTTACCTGTCTC-3’

PGRP1_scrambled		R 5’-AATAAGATGCGGATATTCCACCCTGTCTC-3’
Ixodes scapularis PGRP2_siRNA_44	XM_002433644.1	F 5’-AAGGAATTCCATTGAGCACAACCTGTCTC-3’<br>R 5’-AATTGTGCTCAATGGAATTCCCCTGTCTC-3’
Ixodes scapularis PGRP2_scrambled	N/A	F 5’-AAGCTAGACGCGAACAATATTCCTGTCTC-3’<br>R 5’-AAAATATTGTTCGCGTCTAGCCCTGTCTC-3’
Ixodes scapularis PGRP3_siRNA_151	XM_002410377.1	F 5’-AACCGAATGAGCGTATGATAACCTGTCTC-3’<br>R 5’-AATTATCATACGCTCATTCGGCCTGTCTC-3’
Ixodes scapularis PGRP3_scrambled	N/A	F 5’-AAGCAGTTGAAACGCGATATACCTGTCTC-3’<br>R 5’-AATATATCGCGTTTCAACTGCCCTGTCTC-3’
Ixodes scapularis PGRP4_siRNA_18	XM_002413046.1	F 5’-AAGCTAGGACTGTCTGTCATTCCTGTCTC-3’<br>R 5’-AAAATGACAGACAGTCCTAGCCCTGTCTC-3’
Ixodes scapularis PGRP4_scrambled	N/A	F 5’-AAGCTGTACCGTGCTTATTGACCTGTCTC-3’<br>R 5’-AATCAATAAGCACGGTACAGCCCTGTCTC-3’
Ixodes scapularis MyD88_siRNA_273	XM_002407328.1	F 5’-AAGCATCTCCTCATCCAGAATCCTGTCTC-3’<br>R 5’-AAATTCTGGATGAGGAGATGCCCTGTCTC-3’
Ixodes scapularis MyD88_scrambled	N/A	F 5’-AAGATCATCCCTCGATACACTCCTGTCTC-3’<br>R 5’-AAAGTGTATCGAGGGATGATCCCTGTCTC-3’
Ixodes scapularis JAK_siRNA_743	XM_002406395.1	F 5’-AAGCATCACTTGGACCCACATCCTGTCTC-3’<br>R 5’-AAATGTGGGTCCAAGTGATGCCCTGTCTC-3’
Ixodes scapularis JAK_scrambled	N/A	F 5’-AAGCTTCACACCGAACTACGTCCTGTCTC-3’<br>R 5’-AAACGTAGTTCGGTGTGAAGCCCTGTCTC-3’
Anaplasma marginale msp5 (qRT-PCR)	KP347554	F 5’-CCGAAGTTGTAAGTGAGGG-3’<br>R 5’-CTTATCGGCATGGTCGCC-3’
Dermacentor andersoni actin (qRT-PCR)	EF488512.2[b]	F 5’-CGCCTCCTCCTCTTCTCTG-3’<br>R 5’-TGTAGGTGGTCTCGTGGATG-3’
pCMV-Bendless-FLAG	XM_002400659	F 5’-GAAATTACAAGCTTATGGCCGCCTTACCTAG -3’<br>R 5’-GATTTAAGGGTACCGGCGTTCTGGGCG -3’
pCMV-XIAP-HA	XM_002433822	F 5’-GATTGCAGGAATTCATGGTTGTCATCAGTATGGCG-3’<br>R 5’-GTATAGTTGCGGCCGCTCATGAAAGAAAAGCCTTAATGTTC-3’
pGEX-6P-Bendless	XM_002400659	F 5’-GGATCCATGGCCGCTTTAGGAAG-3’<br>R 5’-CTCGAGTCAGGCGTTCTGGG-3’

[a]Primers were designed based on the *relish* sequence derived from an *I. scapularis* transcriptome (NCBI project number: PRJNA230499).
[b]Primers were designed based on the *D. variabilis actin* sequence*.* The *D. andersoni* product was sequenced and validated as having 100% conserved primer-binding sites.

Supplementary Table 3 (Related to Figure 5). Lipid populations unique to uninfected or *A. phagocytophilum*-infected cells with predicted identities

Uninfected cells *m/z* Observed	*A.p.*-infected cells *m/z* Observed	*A.p.*/Un. FC>2⌘ *m/z* Observed	Predicted Identity	*m/z* Theo.
	730.530		[PC 32:1-H]$^-$	730.5392
	745.469		[PG 34:2-H]$^-$	745.5025
746.504				
		747.489	[PG 34:1-H]$^-$	747.5182
748.504			[PC 34:6-H]$^-$	748.4923
	749.513		[PG 34:0-H]$^-$	749.5338
	758.540		[PC 34:1-H]$^-$	758.5705
	759.506		[PC 34:1+1-H]$^-$ *	759.5182
		773.519	[PG 36:2-H]$^-$	773.5338
		775.542	[PG 36:1-H]$^-$	775.5495
790.511				
837.552			[PI 34:0-H]$^-$	837.5651
	862.711			
889.588			[PI 38:2-H]$^-$	889.5812

⌘ S:N Fold Change (FC)>2, present in both; *A. phagocytophilum* (*A.p*)/cells;

*odd acyl chain PG also predicted by mass, low confidence prediction; database searched for deprotonated ion only [M-H]$^-$; predicted identities reported for error <50ppm.
